# Sponges and Sponge-Like Materials in Sample Preparation: A Journey from Past to Present and into the Future

**DOI:** 10.3390/molecules25163673

**Published:** 2020-08-12

**Authors:** Theodoros G. Chatzimitakos, Constantine D. Stalikas

**Affiliations:** Laboratory of Analytical Chemistry, Department of Chemistry, University of Ioannina, 45110 Ioannina, Greece; cstalika@uoi.gr

**Keywords:** polyurethane foams, melamine sponges, carbon foams, sample preparation

## Abstract

Even though instrumental advancements are constantly being made in analytical chemistry, sample preparation is still considered the bottleneck of analytical methods. To this end, researchers are developing new sorbent materials to improve and replace existing ones, with the ultimate goal to improve current methods and make them more efficient and effective. A few years ago, an alternative trend was started toward sample preparation: the use of sponge or sponge-like materials. These materials possess favorable characteristics, such as negligible weight, open-hole structure, high surface area, and variable surface chemistry. Although their use seemed promising, this trend soon reversed, due to either the increasing use of nanomaterials in sample preparation or the limited scope of the first materials. Currently, with the development of new materials, such as melamine sponges, along with the advancement in nanotechnology, this topic was revived, and various functionalizations were carried out on such materials. The new materials are used as sorbents in sample preparation in analytical chemistry. This review explores the development of such materials, from the past to the present and into the future, as well as their use in analytical chemistry.

## 1. Introduction

Many scientists consider sample preparation as an integral part of the analytical process, which enhances the quality of the obtained results. Others consider sample preparation as the bottleneck of analytical chemistry since labor-intensive steps are required, thus limiting the productivity [1,2]. Nevertheless, more and more emphasis is placed on sample preparation, trying to face the challenges of various matrices in analysis. Since the basic principles of sample preparation remain the same, researchers are developing new sorbent materials to improve and replace existing ones, with the ultimate goal to improve current methods and make them more efficient and effective [3]. 

**Figure 1:**
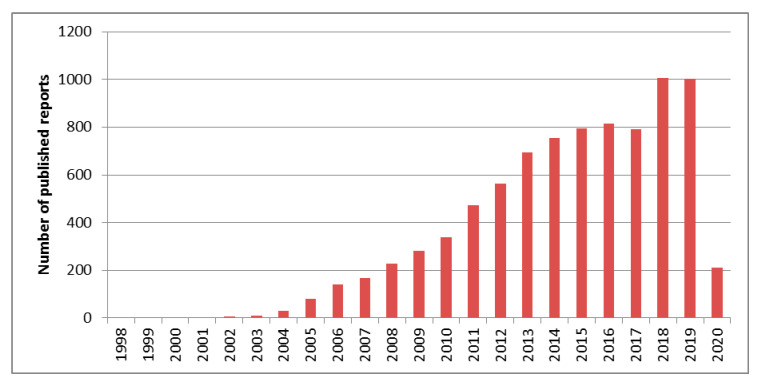
Number of published reports on the use of nanomaterials in sample preparation; source: PubMed.

To this end, back in the 1990s began the use of sponge or sponge-like materials (e.g., foams) in sample preparation [4,5,6]. These materials possess favorable characteristics, such as negligible weight, open-hole structure, high surface area, and variable surface chemistry [7]. As a result, a new trend was started and many reports were published, with the peak reached in the years 1997–2003 [7]. The key merit of these materials that increased their popularity and started the trend was their ability to be compressed into mini-columns so that they could be used under the solid-phase extraction (SPE) principle. Although their use seemed promising, this trend was soon reversed and the number of reports declined significantly. The reasons behind the reversal of the trend are not clear. One possible explanation is the fact that the use of nanomaterials in sample preparation began around the 2000s and, since then, it skyrocketed, as can be seen in Figure 1.

The trend, as detailed below, started with the use of polyurethane foams (PUFs). By definition, PUFs are plastic materials in which gas (in the form of numerous small bubbles (cells)) replace a proportion of the solid phase [8]. Depending on the percentage of the volume the bubbles occupy, the geometrical shape of the bubbles varies from spherical to quasi-spherical polyhedral, thus changing the properties (such as elasticity) of the PUFs. Another important parameter that affects their properties is the synthesis method. PUFs can be fabricated via the reaction either of isocyanates with hydroxyl compounds (resulting in polyester or polyether PUFs) or of isocyanates with water [7]. Either way, the chemical composition of PUFs differs in terms of polar and non-polar groups, thus making them suitable for the sorption of compounds with different properties [9]. While PUFs are the most widely produced foam materials, currently, advancements are being made and materials with similar structure and physical properties are being fabricated. Melamine sponges (MeS) and carbon foams are two typical examples. Melamine sponges are three-dimensional copolymers (composed of formaldehyde, melamine, and sodium bisulfite) with low density, high porosity, and an open-hole structure [10,11]. Their negligible cost and the presence of functional groups, which make them amenable to functionalization, increased their popularity [12]. Likewise, carbon foams are materials with open-cell structures; they have high surface area and tend to be highly hydrophobic [13].

Currently, with the development of new sponge or foam materials, along with the advancement of nanotechnology, the abovementioned topic was revived, and various uses of these materials were proposed. Furthermore, functionalizations were carried out to alter their applicability, so that they could be used as sorbents in sample preparation in analytical chemistry. The recent reports in this field are scanty and sparse, but the use of sponge and foam materials in analytical chemistry is once again an up-and-coming trend. This review explores the development and use of such materials, from past to present, and it highlight futures perspectives on their use in analytical chemistry.

## 2. The Beginning of the Trend

Currently, PUFs are mostly known in analytical chemistry, owing to their widespread use as passive samplers for the collection of volatile compounds [14,15,16,17,18,19]. Their low cost and ease of handling, as well as the fact that they can accumulate particulate matter, make their use favorable. However, PUFs were not always used in such a way. The first reported method that employed PUFs for absorption was published 50 years ago by Bowen, but it was not until the 1990s that the use of PUFs in sample preparation procedures started to become more popular [20]. From that time onward and especially in the next two decades from 1990 to 2010, PUFs were used for the preconcentration of common metal species, such as iron [21,22], copper [23], zinc [6], and nickel [24], and rare metals, such as germanium [4], thorium [25], thallium [26], and uranium [27] from aqueous matrices. In most cases, the PUFs were used bare, without any functionalization, necessitating the addition of organic or inorganic ligands in the sample solution to form metal complexes that could be sorbed onto PUFs. In the case of aqueous samples, no sample pretreatment was carried out, while, for other matrices (such as metal granules, alloys, dried shrimp, fruits etc.), the samples were digested with hot, concentrated nitric acid prior to analysis. A typical example of this concept was the addition of sodium molybdate in a germanium-containing solution so that molybdogermanate could be formed, which was then extracted onto PUFs [4]. The PUFs were left in the sample solution for 1 h (to reach equilibrium). Similar to the above case is the use of polyether-type PUFs for the extraction of gallium (in the form of gallium chloride) from alumina, aluminum alloys, and residues from the aluminum industry [5]. Another example is the use of salicylate for complexation with U^5+^ (in an acidic environment) so that the produced complex could be extracted into PUFs [27]. In all three cases, the PUFs could be analyzed directly, without conducting an elution step, by analyzing the PUFs directly using X-ray fluorescence, which is an asset for the overall time spent for analysis of a sample.

In another study, a method for the determination of molybdenum in iron-based matrices was developed [28]. The method was based on the use of ascorbic acid to reduce Mo^5+^ to Mo^4+^ and Fe^3+^ to Fe^2+^ and then on the employment of thiocyanates to form metal complexes which were extracted into PUFs. The reducing step was important to avoid interferences from iron since Fe^2+^ does not form thiocyanate complexes. The molybdenum thiocyanate complexes were efficiently extracted, even in the presence of ten times as high as the concentration of other metal species (such as copper, cobalt, and zinc), as evidenced by the high recoveries achieved by analyzing pure iron and steel samples. Later on, another study was published, in which the experimental parameters were studied, so that the above-described principle was used for the determination of molybdenum in water and plant leaf samples (digested with the addition of boiling concentrated nitric acid and hydrogen peroxide) [29]. It was found that sorption kinetics were fast since a high flow rate was used (up to 10 mL per min), which was an advantage for the analysis of high sample volumes in a short time. Moreover, it was suggested that the elution should not be carried out with nitric acid solution more concentrated than 3 mol·L^−1^, since the PUF structure is altered and, thus, the PUFs cannot be reused. The recoveries were satisfactory and good accuracy was reported by analyzing certified reference materials. In another study, the use of thiocyanates for the formation of complexes with zinc was proposed, so that they could be extracted onto PUFs [6]. In this method, many metal species, such as calcium, aluminum, and nickel, as well as anions such as chloride, sulfate, nitrate, etc., do not affect the extraction. However, Fe^3+^, Cu^2+^, Co^2+^, Hg^2+^, Ga^3+^, and Pb^2+^ are co-extracted with this method. To avoid their presence, the authors proposed the reduction of Fe^3+^ to Fe^2+^ with ascorbic acid and the use of citrate to mask the copper and cobalt species. The method was developed to extract zinc from cadmium-rich matrices. However, cadmium can also form complexes with thiocyanates. To avoid this, the pH of the solution was adjusted to 3. For the three other metal species, the authors proposed an elution clean-up step with water, which does not elute the mercury, gallium, and lead complexes. The use of water as an elution solvent was also an added advantage since organic solvents were avoided. 

In a similar context, Abbas proposed the use of molybdate for the formation of the respective complexes with phosphates and arsenates, which were then reduced to molybdenum blue (using antimony as a catalyst and ascorbic acid as a reducing agent) and adsorbed into PUFs [30]. The sorbed complexes were then eluted, and the absorbance of the eluent was measured photometrically. However, since both species form molybdenum blue, it was difficult to determine their concentration in the same sample. This is a common problem for the detection of arsenate, which exists at lower concentrations in water samples, compared with phosphates. The author claimed that, by conducting extractions at two pH values (i.e., 0.9 and 1.2, adjusted with sulfuric acid), the formation of molybdenum blue by arsenates was totally inhibited at the solution with pH 0.9 and the recorded absorbance value was only due to phosphomolybdenum blue. Following simple calculations using the two recorded absorbances, the concentrations of both phosphates and arsenates were obtained. Based on the definite formation of the Fe^3+^–thiocyanate complexes, it was proposed that the complexes could be extracted in PUFs [22]. The importance of adding hydrochloric acid (until pH was close to 1.3) to the sample solution so that the formation of Fe^3+^–OH^−^ complexes was avoided was strongly emphasized. The authors proposed that the adsorption of the Fe^3+^–thiocyanate complexes was completed in three steps. Firstly, the solute reaches near the boundary layer film of the adsorbent surface. The second step is film diffusion, which is the diffusion of the complex through the boundary film. The third step is intraparticle diffusion, which is the diffusion of the complex into the porous PUFs. To complete these steps and achieve reproducible results, a 90-min extraction time was proposed. Capitalizing on the same complex formation principle, Casella developed an on-line solid-phase extraction system using PUFs for Fe^3+^ determination in acidic water samples [21]. Following spectrophotometric measurements, highly satisfactory relative standard deviations (between 1.2% and 1.5%) and low detection limits (0.45 or 0.75 μg·L^−1^, depending on the preconcentration time) were achieved. PUFs combined with thiocyanates were also been proposed for the on-line detection of nickel [31]. The use of thiocyanates was suggested, to form complexes with other interfering ions, so that, ultimately, these complexes could be removed by adsorbing into the PUFs, while nickel, which does not form a complex with thiocyanates, could pass through the PUF mini-column. After reacting with 4-(2-pyridylazo)-resorcinol, nickel was determined spectrophotometrically. The two above studies highlighted the potential of PUFs mini-columns for the development of low-cost, on-line systems.

All of the abovementioned studies made use of bare, non-functionalized PUFs. However, there were certain cases where PUFs were loaded with selective reagents for the determination of various ions. This was done to counterbalance two main disadvantages of PUFs: lack of selectivity and low sorption capacity [32]. To make feasible the solid-phase extraction and determination of Ru^3+^, the use of PUFs functionalized with 3-hydroxy-2-methyl-1,4-naphthoquinone-4-oxime was proposed [33]. The developed PUFs were highly selective and made feasible the extraction of 1 μg of Ru^3+^, even in the presence of a high excess of other ions, such as Ba^2+^, Zn^2+^, Cr^3+^, etc. The only metals that could interfere with Ru^3+^ extraction were easily masked with common reagents (e.g., Ni^2+^ was masked by 1% KCN solution; Fe^3+^ and V^5+^ were masked by the addition of one crystal of potassium fluoride and sodium fluoride, respectively). The recoveries were above 98%, highlighting the prospect of functionalized PUFs being exploited. Similarly, the functionalization of PUFs with 9,10-phenanthaquinone monoethylthiosemicarbazone was proposed, so that the functionalization reagent could form a highly stable and colored complex with Ti^3+^ [34]. By using these functionalized PUFs and spectrophotometric detection, recoveries between 99.2% and 100.2% were achieved for zinc granulates and lead foil samples, highlighting the great potential of the method. Similarly, 2-ethylhexylphosphonic acid was proposed as a reagent to functionalize PUFs, to produce selective PUFs for thorium [25]. Thorium ions were extracted based on a cation exchange mechanism. Therefore, highly acidic ambiance was avoided, since hydrogen ions were competitively extracted. Finally, a report was published for the functionalization of PUFs with ammonium hexamethylenedithiocarbamate [35]. The functionalized PUFs were used as sorbents for arsenic, bismuth, mercury, antimony, selenium, and tin. Since, As^5+^, Sb^5+^, and Se^6+^ do not form complexes with ammonium hexamethylene dithiocarbamate, a reduction step was necessary prior to extraction. 

The cases reported so far concerned the extraction of metal ions in (functionalized) PUFs. However, there are two published reports which pertained to the extraction of polycyclic aromatic hydrocarbons (PAHs) in PUFs. In the first study, the authors developed a single-pass flow-through extraction method and examined the suitability of PUFs for the extraction of 18 PAHs from diesel exhaust samples [36]. In the second study, PUFs were suggested for the extraction of four PAHs from water samples [37]. The authors examined the mechanism via which the PAHs and PUFs interact. Firstly, they found that PAH sorption was not dependent on the chemical structure of the PUFs. Whether the polyester, the polyether, or their co-polymer was used, sorption remained unchanged. However, it was evident that the hydrophobicity of the PUFs greatly influenced their sorption potential. A more hydrophobic PUF presented a greater sorption potential. Both of these studies highlight the effectiveness of bare PUFs for PAH extraction, without laborious functionalization steps. A summary of the analytical methods developed, between 1990 and 2010, based on PUFs is given in Table 1.

## 3. The Revival of the Trend

### 3.1. Recent Uses of Polyurethane Foams

After a marked decline in the number of publications with the use of sponge and sponge-like materials in sample preparation, after 2010, this trend was revived, and more and more studies were published. As with the reports of the previous years, some PUF-based sorbents were developed for metal species, albeit to a lower extent [9,32,57,58]. For instance, the use of Eriochrome Black T as a complexing agent for Cu^2+^ was recently proposed [9]. Eriochrome Black T was selected since, in acidic conditions, the metal–Eriochrome Black T complex formation constants for copper and iron are higher than those for other metals (such as cobalt, zinc, etc.). The formed complexes were sorbed on PUFs and, based on this, an SPE procedure was developed for Cu^2+^ extraction from water samples. At acidic ambiance, the copper–Eriochrome Black T complex was in its neutral form and was extracted more efficiently. This was justified by proposing a solvent-like mechanism, where PUFs acted as a polymeric solvent, which was able to retain neutral substances or substances with very low charge density. Although Eriochrome Black T forms more stable complexes with iron than with copper, the iron complexes could not be sorbed efficiently on PUFs, because, at acidic ambiance, nitrogen atoms of PUFs are protonated and repulse the cationic iron–Eriochrome Black T complex. Capitalizing on the affinity of thializodin-4-ones for heavy metal ions, a spirothializodine analogue (3-sulfonamoyl-phenyl-spiro[4-oxo-thiazolidin-2,2′steroid]) was synthesized to functionalize PUFs with it so that PUFs were rendered selective and able to preconcentrate Cd^2+^ from water samples containing iodide ions [57]. Iodide ions were added to the sample solution so that the anionic complex [CdI_4_]^2−^ could be formed, which formed a ternary ion that could be sorbed onto PUFs. In another study, an SPE procedure for the separation of Au^2+^ traces from geological samples was developed [32]. To make this feasible, acid hydrolysis of the PUFs was carried out, and then, using glutaraldehyde as a linking arm, cytosine was added onto the PUFs. Cytosine was selected due to its low cost and its nitrogen and oxygen atoms that render it a good ligand for metal ions. To carry out the extraction, the pH of the sample solution was adjusted to 1 so that protonation of the binding sites of the chelators could take place, while avoiding metal precipitation from hydroxides. The use of hydrochloric acid ensured the formation of the chloro-anionic species (AuCl_4_^−^) which are readily adsorbed onto the imine groups of the cytosine-modified PUFs, via electrostatic interactions (formation of ion-pair complexes). The functionalized PUFs exhibited high selectivity, sensitivity, and high adsorption capacity for Au^2+^. PUFs were also functionalized with β-naphthol and used as sorbents for Fe^3+^, Cu^2+^, Cr^3+^, Co^2+^, and Mg^2+^ [58]. The developed PUFs efficiently adsorbed the metal species, following a simple SPE procedure. In the case that methylene blue was used to functionalize PUFs, penicillins could be extracted from pharmaceuticals and milk samples (previously deproteinated with the addition of acetic acid), following a simple SPE procedure [59]. The developed procedure was sensitive to pH changes since they affected the formation of ion pairs between the antibiotics and the methylene blue-functionalized PUFs. At pH values lower than 8, antibiotics are primarily in their neutral form; hence, they cannot form ion pairs. At pH above 9.5, a competition between the antibiotics and the hydroxyl ions for the positively charged centers of the sorbent was recorded. The method developed exhibited adequate accuracy and good precision. More importantly, the reusability of the prepared PUFs was examined, and it was found that they can last six months, after performing 15 sorption–desorption cycles each day, which is a great asset of the developed material. 

Finally, PUFs were functionalized with graphene oxide (GO) to combine the extractive properties of both [60]. The preparation process is very easy (stirring PUFs in GO solution and then drying at room temperature), but it takes more than 24 h to be completed. The epoxy groups of the GO link with the carboxyl and amino groups of the PUFs, while the formation of hydrogen bonds further stabilizes the coating. The prepared sponges were used to extract sulfonamides from milk samples (after protein precipitation with acetonitrile). From analyzing the Fourier-transform infrared (FT-IR) spectra of the sorbent prior and after the extraction, the authors found that amides where formed, as a result of the sulfonamides amine groups and the carboxylate groups of the sorbent. Moreover, the formation of hydrogen bonds was validated and contributed to the overall sorption of sulfonamides. A summary of the analytical methods, developed between 2005 and today, based on PUFs is given in Table 2.

### 3.2. Development of Carbon-Based Foams

**Figure 2:**
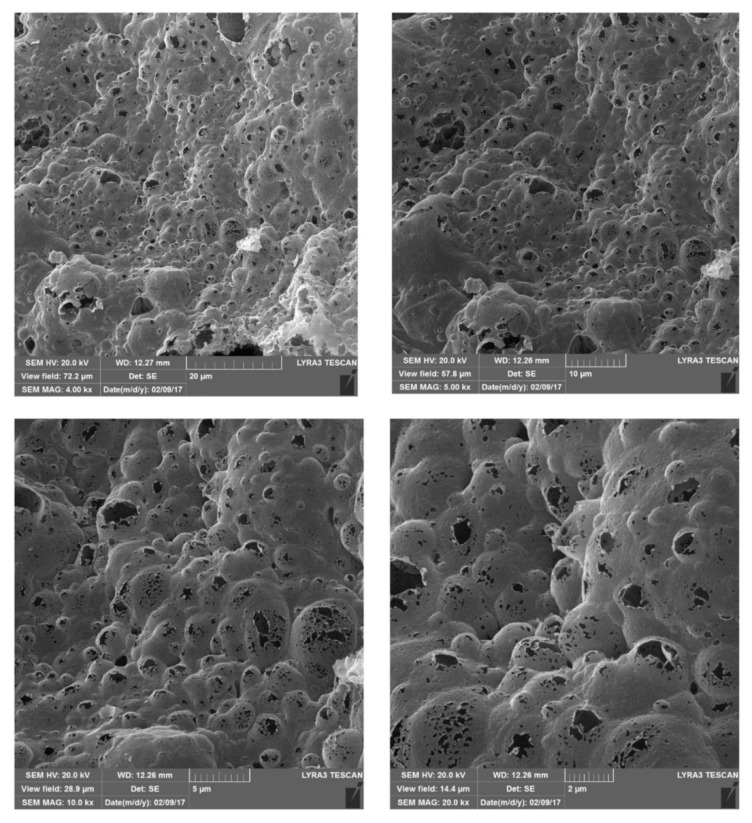
Field-emission (FE)-SEM images of carbon foam. Source: Reproduced from [65], with permission from Elsevier.

All the above studies highlight the potential of PUFs in sample preparation. Currently, the trend seems to have shifted toward other sponge-like materials. One such material is carbon-based foam. In one study, melamine–formaldehyde polymer foams were annealed at 800 °C, under a nitrogen atmosphere, to produce carbon foams [61]. The obtained carbon foams retained the initial three-dimensional (3D) interconnected network of the initial foams, and they were composed of nitrogen and carbon atoms, while exhibiting moderate hydrophobicity. If the temperature during synthesis was increased to more than 1000 °C, more hydrophobic carbon foams would be obtained. The carbon foams were used for the extraction of phenolic endocrine-disrupting compounds (i.e., bisphenol A, 4-*tert*-octylphenol, and 4-*n*-nonylphenol) from water samples, since they have a good affinity for moderately polar phenols, owing to their hydrophobicity. The recoveries were found to be above 90% for experiments conducted using well water, leachates, and wastewater. The use of the synthesized carbon foams resulted in enhanced preconcentration factors (i.e., 24–38), compared to PUFs (preconcentration factors: 11–15) and MeS (preconcentration factors: 7–12). In two other studies, GO/polypyrrole foams were developed and used in pipette-tip SPE procedures [62,63]. In the first case, the polypyrrole (by polymerization of pyrrole in the presence of FeCl_3_) was firstly prepared and then mixed with a GO solution for 24 h [62]. Although the final material was obtained in the form of a powder, the scanning electron images revealed that the morphology was loose three-dimensional foam. The GO/polypyrrole foams were used to extract three auxins from papaya juice. In the second case, the polymerization of the pyrrole was conducted in the presence of GO and, thus, the amount of time needed for the preparation of the GO/polypyrrole foams was significantly reduced (from 48 h needed in the previous case to 12 h) [63]. The above GO/polypyrrole foams were used to extract seven sulfonamides from milk and honey samples. Acetonitrile and hexane were successively added to the samples to remove proteins and fat, respectively. The proposed method consumes a very small amount of adsorbent (3 mg) and the extraction step is completed in 3 min, which are significant assets of the developed method. The relative standard deviations (RSDs) achieved with the proposed method were low (<1.1% for intra-day analyses and <1.9% for inter-day analyses) when analyzing water samples. However, the recoveries were less satisfactory for real samples (62.3–109.0% and 66.6–106.9% for honey and milk samples, respectively) and the RSDs were significantly higher (<11.2% and <10.8% for honey and milk samples, respectively). Despite the significant advantages of the method, further improvements are needed to enhance its performance. Another case was the freeze-drying of a GO dispersion to obtain a GO sponge which was reduced to form a graphene sponge, by following a reduction step using hydrazine [64]. The SEM images revealed that the GO sponge had a more compact structure, compared to graphene sponge, which was attributed to the interactions of the oxygen-containing groups. In both cases, a smooth structure and a porous three-dimensional open-hole structure were observed. Using the graphene sponges under the principle of solid-phase extraction, the authors were able to extract six organic ultraviolet (UV) filters (i.e., 2-(2′-hydroxy-5′-methylphenyl) benzotriazole, 2-(2*H*-benzotriazol-2-yl)-4,6-bis(1-methyl-1-phenylethyl)phenol, 2-*tert*-butyl-6-(5-chloro-2*H*-benzotriazol-2-yl)-4-methylphenol, 2-(2′-hydroxy-3′, 5′-di-*tert*-butylphenyl)-5-chlorobenzotriazole, 2-(2′-hydroxy-3′,5′-dipentylphenyl) benzotriazolel, and 2-(2*H*-benzotriazol-2-yl)-4-(1,1,3,3-tetramethylbutyl)phenol) from water and personal care products (e.g., skin cream, sunscreen etc.). A wide linear range (20.0 to 1000 μg·L^−1^), was recorded for the developed method. The synthesized graphene sponges could be reused more than 60 times, which counterbalances their lengthy preparation (five days are needed to prepare 4.5 g of graphene sponge, starting from pristine graphite oxide to synthesize GO and then the sponges). In another study, the authors heated a mixture of zinc nitrate and sucrose in a crucible at 120 °C for 2 min, then at 180 °C for 5 min, and then at 1100 °C for 3 h, combining synthesis and calcination in a single step [65]. During the heating step, various gases are released (such as CO_2_, N_2_, and H_2_O vapors) that “blow” the heated mixture and form the foam-like structure. The SEM images revealed that the foam material contains many “bubbles”, some intact and others broken, which facilitate the interaction of the foam material with analytes, not only on the surface but also in the internal holes, cavities, and channels (Figure 2). The carbon foam developed was used in a stir-bar-supported micro-solid-phase extraction procedure for the extraction of five polyaromatic hydrocarbons from wastewater samples. The performance of the carbon foam was similar to that of multiwalled carbon nanotubes and graphene; however, since its synthesis is faster and cheaper, it is a good alternative to the other two nanomaterials. A summary of sample preparation procedures developed based on various carbon-based foams is given in Table 3.

### 3.3. Combinations of Carbon-Based Foams with Metals

**Figure 3:**
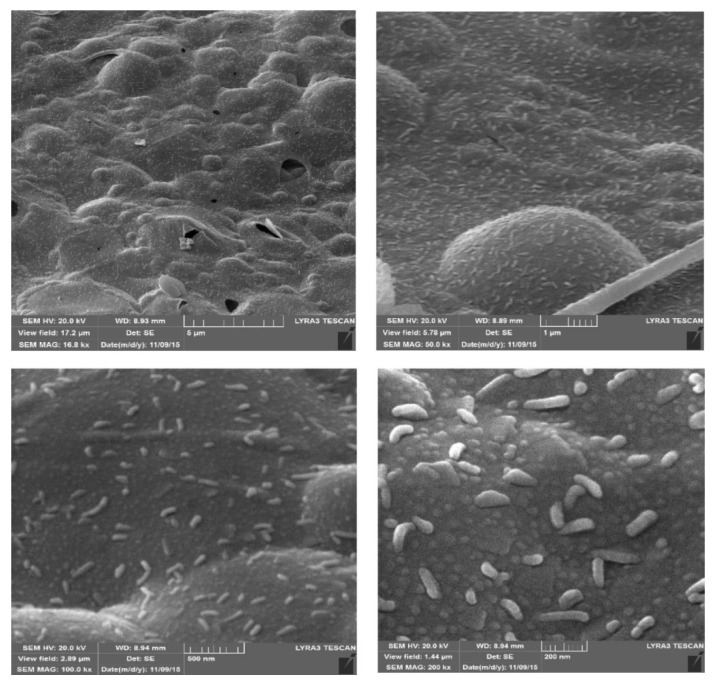
SEM images at different magnifications showing the distribution of ZnO nanoparticles over the surface of carbon foam. Source: Reproduced from [12], with permission from Elsevier.

Instead of using solely carbon-based foams, in some cases, carbon-based foams functionalized with metals were developed. One good example is the study published by Sajid et al., who prepared carbon foam with zinc oxide nanoparticles incorporated in the network [13]. To do so, they heated a mixture of sucrose and zinc nitrate at 110 °C. The concept is similar to that discussed above. However, in this case, an annealing step was not employed. The resulting product had a foamy structure, as evidenced by scanning electron microscopy images, while zinc oxide nanoparticles were visible all over the surface (Figure 3). The presence of zinc oxide nanoparticles was further confirmed by X-ray diffraction (XRD) spectra. The authors also calcinated the produced zinc oxide nanoparticle-incorporated carbon foam by heating at 900 °C. Although the calcined product exhibited a better crystalline structure, its sorption performance was worse, compared with the as-synthesized foam. The developed product was used as a sorbent for the extraction of 15 organochlorine pesticides from milk samples (without sample pretreatment). In another study, the authors combined chitosan and metal–organic frameworks to prepare foams [66]. The foams were prepared by an ice-templating procedure where proper amounts of the metal–organic framework, chitosan, and glutaraldehyde were mixed, and then the mixture was placed into a mold. After freezing at −20 °C, the material was freeze-dried to form the porous foams. The authors prepared six such foams, using different metal–organic frameworks (i.e., MIL-53(Al)/chitosan, MIL-53(Fe)/chitosan, MIL-101(Cr)/chitosan, MIL-101(Fe)/chitosan, UiO-66(Zr)/chitosan, and MIL-100(Fe)/chitosan), and they examined their sorptive performance for a mixture of five parabens in water. The results were conclusive that the best sorbent was MIL-53(Al)/chitosan foam.

Metal–organic frameworks exhibit drawbacks in aqueous-phase adsorption due to low stability in water (coordination bonds are likely to collapse). For this reason, the use of a zeolitic imidazolate framework-8 (ZIF-8) in combination with a GO sponge was proposed [67]. An ice-templating procedure was employed to obtain a product, similar to the previous case; however, to obtain a functional material, a calcination step at 800 °C was necessary. Without the calcination step, the material had a mono-dispersion rhombic dodecahedral structure, similar to that of ZIF-8. When the material was calcined at 800 °C, a rich open-hole structure could be observed, which could not be achieved at lower temperatures. The ZIF-8/GO sponge was used for the extraction of five sex hormones in defatted and deproteinated milk and milk products. The developed method exhibited wide linear ranges (10.0–3000 mg·L^−1^) with remarkable linear correlation coefficients (*R*^2^ > 0.9998). Excellent repeatability (intra-day RSDs < 0.39% and inter-day RSDs < 3.86%) and good recoveries (83.8–108.4%) were the two most significant advantages of the developed procedure. Finally, in another study, the authors employed a somewhat “reversed” concept, where, instead of functionalizing a carbon-based foam with some metal, they functionalized nickel foams with polydopamine [68]. This was achieved by placing nickel foam in a dopamine solution prior to its self-polymerization. Owing to the presence of catechol and quinine groups on the surface of the prepared foam, good affinity with Sudan dyes was expected. As a proof of concept, the developed foam was used in a solid-phase microextraction procedure for four Sudan dyes from diluted tomato sauce and hotpot seasoning samples. A summary of the analytical methods developed based on carbon-based foams functionalized with metals is given in Table 4. 

### 3.4. Development of Functionalized Melamine Sponges

**Figure 4:**
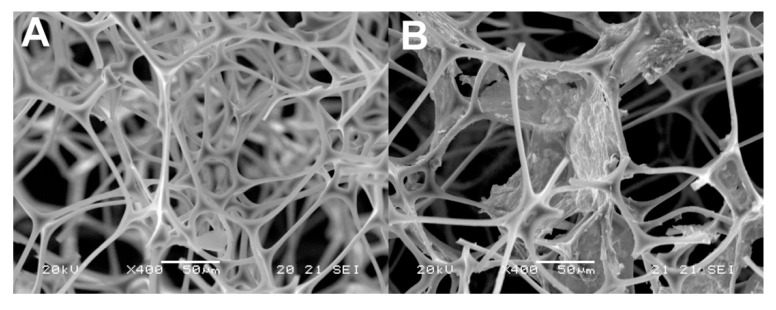
SEM images of MeS before (**A**) and after (**B**) functionalization with graphene (GMeS). Source: Reproduced from [10], with permission from Elsevier.

**Figure 5:**
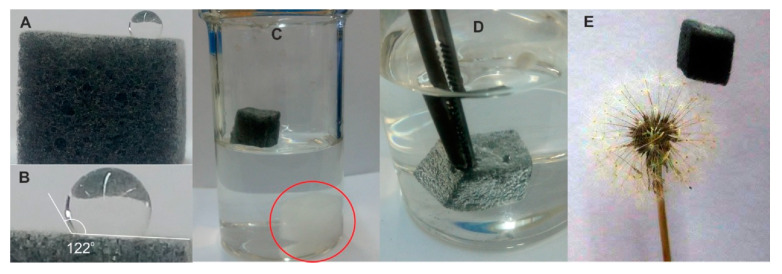
Images of (**A**) water droplet on the surface of a GMeS; (**B**) contact angle of a water droplet; (**C**) MeS and GMes in a glass beaker with water; (**D**) GMeS immersed in water; (**E**) GMeS on top of a dandelion flower. Source: Reproduced from [10], with permission from Elsevier.

**Figure 6:**
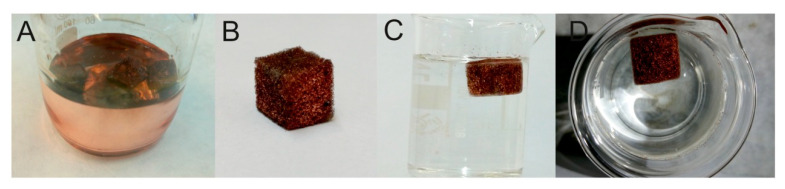
Copper mirror formed on the surface of the reaction glass beaker during the decoration of MeS (**A**). The produced CuMeS (**B**) and its behavior in water (**C**,**D**). Source: Reproduced from [11], with permission from Elsevier.

Until recently, MeS was an unexplored material in sample preparation. In our laboratory, we modified MeS with graphene (GMeS) in a straightforward way and used it, for the first time, in sample preparation [10]. Previous studies attempted to modify MeS with graphene, using multiple steps and sophisticated equipment, resulting in time-consuming methods. We achieved the modification by dipping MeS cubes into a GO solution, containing hydrazine, before irradiating the cubes with microwaves for 2 min and then drying. The as-prepared GMeS contained G sheets through their structure (Figure 4) and were rendered hydrophobic (Figure 5). The prepared GMeS were used for the extraction and preconcentration of sulfonamides from deproteinated milk and eggs, as well as lake water samples (based on π–π and hydrophobic interactions), and a method validated according to the SANCO/12571/2013 guideline was developed. Low limits of quantification (between 0.31 μg·kg^−1^ and 1.3 μg·kg^−1^ for the food samples and between 0.10 μg·L^−1^ and 0.29 μg·L^−1^ for lake water samples), and high enrichment factors for milk and lake water samples (94–100) were some of the figures merit of the developed procedure. Following this study, next, we proposed the decoration of MeS with copper sheets, so as to prepare a sorbent, selective and suitable for sulfonamide extraction, based on the affinity of copper for sulfonamides [11]. The synthesis was based on the addition of hydrazine in a heated solution of copper acetate, in which MeS was placed, and stirring the mixture for 30 min (Figure 6). After washing, the copper-decorated MeS could be used, directly, without the need for a drying step. Owing to the size of the copper-decorated MeS, sulfonamides could be extracted following a radically different mechanism. Their sorption was based on the fact that sulfonamides acted as bridges between two copper ions, via their aromatic amine nitrogen and the nitrogen atom of the heterocycle. This mechanism renders the sorbent selective and efficient for sulfonamides. Using the prepared sponges, we developed a method for sulfonamide determination in deproteinated milk and water samples, validated according to the Commission Decision 657/2002/EC. The method exhibited a wide linear range (0.05–150 μg·L^−1^) and high enrichment factors (234–463 for water samples), which render it suitable for the routine analysis of sulfonamides. In another study, we functionalized MeS with urea–formaldehyde co-oligomers [12]. Instead of adopting an acid-catalyzed polymerization step for the preparation of urea–formaldehyde oligomers, we employed a base-catalyzed step. This resulted in the formation of a more hydrophobic product, which does not exhibit the typical resin structure of the acid-catalyzed polymer and consists mainly of oligomers. The prepared sponges were found to be suitable for the extraction of six different classes of compounds (i.e., non-steroidal anti-inflammatory drugs, benzophenones, parabens, phenols, pesticides, and musks). The developed method had low limits of quantification (0.03 and 1.0 μg·L^−1^), wide linear ranges, and excellent recoveries. 

In another study, graphene-modified MeS were functionalized with β-cyclodextrin [69]. The synthesis procedure consisted of multiple steps: Firstly, MeS was dipped into a GO solution and dried. Then, the sponges were placed into a solution of β-cyclodextrin (previously modified with aminopropyl tetraethoxysilane), removed after 2 h, and left to dry overnight. The modification procedure was repeated once more, resulting in the final material. The sponges were used for the extraction of flavonoids. The developed material is new and has some advantages, but the synthesis is time-consuming. MeS functionalized with β-cyclodextrin and graphene was also proposed [70]. An MeS cube was added into a β-cyclodextrin and graphene dispersion, and then ammonia solution was added to adjust pH to 10. After adding hydrazine and heating, the modified MeS was dried. The developed sponges could be used for the extraction of malachite green. The presence of β-cyclodextrin was found to significantly affect the adsorption, since sponges prepared with lower amounts of β-cyclodextrin had lower sorption efficiency. The sponges were employed to extract malachite green from fresh crayfish and squid extracts (samples were homogenized and acetonitrile was added to extract malachite green). Another type of functionalization for MeS reported was the use of carboxylated multi-walled carbon nanotubes and the metal–organic framework MIL-101(Cr) [71]. The synthesis was based on mixing carboxylated multi-walled carbon nanotubes, MIL-101(Cr), and polyvinylidene difluoride, and then immersing MeS into the final solution. The modified sponges were used in an SPE procedure for the extraction of six triazines from corn extracts (corn was crushed to fine powder, and hexane was used to extract the compounds). 

To coat MeS with polyaniline a new procedure was developed [72]. Since polyaniline is a polymer with four different states, it can serve well as a sorbent in sample preparation. The synthesis of the sponges was very simple. After dipping the MeS into an aniline solution and freezing them at 4 °C for 30 min, a chilled solution of ammonium persulfate was added, and the mixture was stirred for 30 s. Then, the mixture was left at 4 °C for 4.5 h and, after rinsing and drying, the sponges were ready to be used. Stirring was avoided during synthesis so that the formation of polyaniline agglomerates in the MeS could not be present. The modified MeS was found to be suitable for the extraction of perfluorooctanoic acid and perfluorooctane sulfonate from deproteinated (with acetonitrile) human urine and serum. A similar simple procedure was followed in another study, were silanization of MeS with trichloromethylsilane was completed in 10 min [73]. The sponges were rendered hydrophobic after the silanization, which made it suitable for the extraction of benzene, toluene, ethylbenzene, *m*-xylene, and *o*-xylene. The adsorption was based mainly on hydrophobic interactions, since benzene was adsorbed less efficiently than *m*-xylene (K_o/w_ for *m*-xylene is 10 times higher than that of benzene). Following a needle-trap extraction method, an analytical procedure was developed which exhibited low limits of detection (0.005–0.0010 μg·L^−1^). In a more complex study, layered double hydroxides were developed on the surface of MeS [74]. To do so, the Co(II)/2-methylimidazole porous coordination polymers were firstly immobilized on MeS and served as a source of Co, so that Ni–Co layered double hydroxides could be synthesized on the MeS. Three phenolic acids (gallic, *p*-hydroxybenzoic, and caffeic acid) were used to examine the suitability of the developed sponges in sample preparation. It is noteworthy that the developed layered double hydroxides were dissolved during the elution step to obtain the analytes. Owing to the good analytical figures of merit, a simple and effective analytical method was developed. Compared to the extraction with bare layered double hydroxides, the composite material had superior performance, due to the increased surface area. This is probably one of the reasons that the use of sponges and sponge-like materials is again a trend, since the surface area of existing compounds can be increased, by “depositing” them onto sponges. Finally, Liu et al. formed silica monoliths on the surface of MeS [75]. The sponges were dipped into a hydrolyzed mixture of tetramethoxysilane and vinyltrimethoxysilane, containing polyethylene glycol, urea, and acetic acid, and then heated. To render the sponges suitable for the extraction of dipeptides, sponges were modified with 3-mercapto-1-propanesulfonic acid so that sulfonate groups could interact with the free amine groups of the peptides. The silica-monolith-functionalized MeS could also be easily used for other applications, by altering the synthesis mixture. For instance, the addition of β-cyclodextrin during synthesis makes the sponges effective for the sorption of 4,4′-sulfonyldiphenol. Analytical methods developed based on modified melamine sponges are summarized in Table 5. 

### 3.5. Use of Natural Sponges

The use of natural sponges has many benefits, including renewability, low cost, environmental friendliness, etc. Therefore, the use of natural sponges is highly promising. It is known that sea sponges are used as biomarkers to monitor the contamination of water with heavy metals [76]. Therefore, they are suitable for the preconcentration of metals. Capitalizing on this principle, a sea sponge was used to fill a column, which was used to extract copper, iron, lead, and nickel [76]. Due to the complex composition of the sponge and the plenty functional groups, no complexation agents were needed to sorb the metals, as in the case of PUFs. Moreover, the developed method is environmentally friendly, as it uses natural sea sponge. Later, the same groups published another study, where they proposed the use of sea sponges for the adsorption of Ponceau 4R and Sudan Orange G dyes [77]. The sea sponges served as an excellent sorbent since very good analytical figures of merit were recorded and, more importantly, very good recoveries were obtained from analyzing real samples without any pretreatment (i.e., peach-flavored drink powder, fruit-flavored mint candy, flavored rock candy, rosehip-flavored drink powder, fruit-flavored soft drinks, tomato paste, and pepper). No interference was recorded from ions commonly present in food products (such as iron, copper, potassium, calcium, etc.) or other food dyes (chocolate brown, tartrazine, sunset yellow, brilliant blue, patent blue V), even though, in their previous study, they found that metals could readily be adsorbed from the sea sponges. These two studies highlight the potential of sea sponges in sample preparation.

*Luffa* sponge is another natural sponge, obtained from the ripened fruits of *Luffa cylindrica* [78]. It is composed of lignin, cellulose, hemicellulose, and smaller quantities of pectin and proteins. Owing to its many functional groups, it can also serve as an excellent sorbent material. This is evidenced by three recent reports. In the first report, *Luffa* sponges were used as a sorbent for phosphopeptides from protein digests [78]. The sponges exhibited exceptional selectivity for phosphopeptides, over other non-phosphopeptides, making feasible their detection, even when their concentration was 100 times lower than other non-phosphopeptides. In the second report, the authors used *Luffa* sponges for the selective extraction of chromium (III) [79]. Selectivity over chromium (VI) was ensured by adjusting the pH of the solution to 4.0. The analytical method developed exhibited high accuracy as evidenced by analyzing certified reference materials (certified value: 300.00 ± 0.5, found value: 299.57 ± 0.006). In the final report, the ionic liquid 1-hexadecyl-3-methylimidazolium bromide was deposited on *Luffa* sponges by physisorption [80]. The modification was carried out to render *Luffa* sponges suitable for the determination of four benzoylurea insecticides in water and tea beverage samples. Before the addition of the ionic liquid, an alkali treatment of the *Luffa* sponges was carried out to remove hemicellulose and lignin and to make the sponges more hydrophilic. 

## 4. Conclusions

Herein, we discussed the use and selected applications of sponges and sponge-like materials in analytical chemistry. This trend started with the use of PUFs for metal ions sorption. Although quite a few articles were published on this topic due to the limitations of PUF use, the narrow applicability (only metal ions), and the advancement of nanotechnology, this trend soon declined. However, in the last decade, this trend was not only reversed, but more and more researchers also aimed to develop new sorbent materials, based on sponges. Currently, many different sponge materials exist, such as PUFs, MeS, carbon foams, sea sponges, *Luffa* sponges, and others. This makes it easier to develop more advanced or selective sorbents than in the past. The next step in this field was the combination of nanomaterials with sponges. This resulted in the development of sorbents with more advanced characteristics and wider sorption capabilities, rendering sponges suitable for the sorption of small organic molecules, such as antibiotics and pesticides. In the future, large-scale synthesis of the materials should be examined, so as to result in commercially available and reliable sorbent materials. Moreover, the use of other nanomaterials should also be examined in order to make even wider the gamut of potential sorbents, as well as to render them more suitable for specific applications. Whether this trend will come to its own in sample preparation or not remains to be seen in the near future. Until then, the development of sponge-based sorbents will continue to improve, resulting in exceptional materials that could significantly alter existing methods.

## Figures and Tables

**Table 1:** Summary of the analytical methods developed, between 1990 and 2005, based on polyurethane foams (PUFs), SP: spectrophotometry; ETAAS: electrothermal atomic absorption spectrometry; XRF: X-ray fluorescence spectrometry; AAS: atomic absorption spectrometry; ICP-AES: inductively coupled plasma atomic emission spectrometry; PAH: polycyclic aromatic hydrocarbon.

Analyte	Ligand	Sample	Extraction Time (min)	Analytical Technique	LOD (Limit of Detection) μg·L^−1^	Reference
Ge	Molybdate	Water	60	XRF	70	[4]
Zn^2+^	Thiocyanate	Cadmium-rich matrices	10	SP	20	[6]
I, Hg, Au, Fe, Sb, Th, Mo, Re, U, benzene, chloroform, phenol	-	Water	30	SP	-	[20]
Fe^3+^	Thiocyanate	Water and rice flour	50	SP	450	[21]
Fe^3+^	Thiocyanate	Water	5	SP	-	[22]
Cu^2+^	Diethyl dithiocarbamate	Water	40	ETAAS	-	[23]
Zn^2+^	Thiocyanate	Aluminum matrices	10	ICP-AES	0.02	[24]
Th	2-Ethylhexylphosphonic acid	Water	30	XRF	4.0	[25]
U	Salicylate	Water	50	XRF	5.5	[27]
Mo	Thiocyanate	Steel and pure iron	10	ICP-AES	0.9	[28]
Mo	Thiocyanate	Water, peach, apple, and citrus leaves	-	ETAAS	0.08	[29]
AsO_4_^3−^ and PO_4_^3−^	Molybdate	Water	-	SP	5.4 and 1.66	[30]
Ni	Thiocyanate	Silicates and alloys	-	SP	77	[31]
Ru^3+^	3-Hydroxy-2-methyl-l,4-naphthoquinonexime	Water	3–5	SP	20	[33]
TI^1+^ and TI^3+^	9, 10 Phenathaquinone monomethyl thlio semicarbazone	Water and lead solutions	30	SP	2.76	[34]
As, Bi, Hg, Sb, Se, Sn,	Dithiocarbamate	Water	-	ETAAS	0.06–0.3	[35]
PAHs	-	Diesel exhaust		GC/MS	-	[36]
PAHs	-	Water	30	Solid-matrix spectrofluorimetry	0.02	[37]
Cu^2+^	-	Dried shrimp		SP	1.2	[38]
Co, Fe, Zn, Cd, Ni, Hg	Thiocyanates (for Co, Fe, Zn), 1-(2-pyridylazo)-2-naphthol (for Ni, Hg, Cd)	Water	180	SP	-	[39]
Co^2+^	thiocyanate	Water	5	SP	-	[40]
PAHs	-	ambient air	960	GC/MS	-	[41]
As, Bi, Pb, Sb, Sn, Se, Hg	Dithiocarbamate	Waste Water and seawater	60	ICP-AES	0.03–30	[42]
chlorobenzenes		ambient air	1320	GC/MS	-	[43]
Two-ring aromatic hydrocarbons, chlorinated phenols, guaiacols, and benzenes	-	Ambient air	720	GC/electron capture detection	200–500	[44]
Cd, Co, Cu, Hg, Ni, and Pb	Hexamethylene ammonium hexamethylene dithiocarbamate	Oxalic acid	-	AAS or ICP-AES	0.1–0.3	[45]
Zn	Thiocyanate	Water	5	SP	0.9	[46]
Ni^2+^	Dimethylglyoxime	Water	15	SP	0.5	[47]
acaricides	-	Water	10	SP	-	[48]
Co^2+^	Thiocyanate	Water	2	Gamma-spectrometer	-	[49]
Al	Thiocyanate	Rhyolite, syenite, andesite, basalt, iron ore	30	SP	30	[50]
Dimethote, azodrine, lannate	-	Water	10	SP	-	[51]
As	-	Water	60	XRF	36	[52]
Polychlorinated biphenyls, PAHs, and n-alkanes	-	Diesel exhaust, cigarette smoke, and roofing tar volatiles	<20	GC/MS	-	[53]
Ag	-	Water	20	SP	-	[54]
Ascorbic acid	Molybdosilicic heteropolyacid	Fruit juices and pharmaceutical preparations	6.5	SP	0.6–40	[55]
Zn, Hg, In	Thiocyanate	Water	30			[56]

**Table 2:** Summary of the analytical methods developed, between 2005 and the present, based on PUFs. HPLC-UV: high-performance liquid chromatography with ultraviolet detection.

Analyte	Functionalization Agent	Sample	Extraction Time (min)	Analytical Technique	LOD (Limit of Detection) μg·L^−1^	Reference
Cu^2+^	Eriochrome Black T	Water	30	Flame atomic absorption spectrometry	20–100	[9]
Cd^2+^	3-Sulfonamoyl-phenyl-spiro[4-oxo-thiazolidin-2,2′steroid]	Industrial wastewater	20	Flame atomic absorption spectrometry	30–100	[57]
Fe^3+^, Cu^2+^, Cr^3+^, Co^2+^, and Mn^2+^	β-Naphthol	Water		Flame atomic absorption spectroscopy		[58]
Penicillin G, amoxicillin, and ampicillin	Methylene blue	Pharmaceuticals and milk	-	Flow injection analysis/solid-phase extraction	12, 15, and 19	[59]
Sulfathiazole, sulfamethizole, sulfadiazine, and sulfanilamide	Graphene oxide	Cow milk	15	HPLC-UV	50	[60]
Au^3+^	Cytosine	Geological samples	-	Inductively coupled plasma optical emission spectrometry	0.006	[32]

**Table 3:** Analytical methods developed based on carbon-based foams. RSD: relative standard deviation.

Precursors for Carbon Foam	Analyte	Sample	Analytical Technique	LOD (μg·L^−1^)	Recoveries (%)	RSD (%)	Reference
Carbonization of melamine sponges	bisphenol A, 4-*tert*-octylphenol, and 4-*n*-nonylphenol	Well water, rainwater, and wastewater	Sequential injection analysis	0.02–0.04	>89.0	2.8–6.3	[61]
Graphene oxide/polypyrrole	Sulfathiazole, sulfapyridine, sulfamethizole, sulfadoxine, sulfisoxazole, sulfamethoxazole, and sulfadimethoxine	Honey and milk	HPLC-UV	0.00104–0.00150	62.3–109.0	>11.2	[63]
Graphene oxide/polypyrrole	Indole-3-butyric acid, indole-3-propionic acid, and 1-naphthaleneacetic acid	Papaya juice	HPLC-UV	0.0012–0.0017	89.4–105.6	<3	[62]
Graphene oxide	Organic UV filters (UV-P, UV-234, UV-326, UV-327, UV-328, and UV-329)	Water and cosmetic products	HPLC-UV	0.02–0.08	89–105	<8.1	[64]
Zinc nitrate and sucrose	Naphthalene, biphenyl, acenaphthene, fluorene, and phenanthrene	Wastewater	GC/MS	0.29–8.4	91.8–102	3.8–10.9	[65]

**Table 4:** Analytical methods developed based on carbon foams with metals.

Sorbent	Analyte	Sample	Analytical Technique	LOD (μg·L^−1^)	Recoveries (%)	RSD (%)	Reference
Zinc oxide-incorporated carbon foam	Organochlorine pesticides	Milk	GC/MS	0.19–1.64	85.1–100.7	2.3–10.2	[13]
Metal organic framework/chitosan foams	Parabens	Water	UPLC–MS/MS	0.09–0.45	78.75–102.1	<7.4	[66]
Zeolitic imidazolate framework-8@graphene oxide sponge	Sex hormones	Milk and milk products	HPLC	520–2110	83.8–108.4	<0.39	[67]
Nickel foam functionalized with polydopamine	Sudan dyes	Tomato sauce and hotpot sample	Ion mobility spectrometry	0.005–0.25	81%–91.3	<15.5	[68]

**Table 5:** Analytical methods developed based on melamine sponges. MWCNT: multi-walled carbon nanotube.

Functionalization Moieties	Analyte	Sample	Analytical Technique	LOD (μg·L^−1^)	Recoveries (%)	RSD (%)	Reference
β-Cyclodextrin/graphene oxide	Flavonoids	*Lycium barbarum*	HPLC	0.5–2	77.9–102.6	3.5–6.8	[69]
Polyvinylidene difluoride-MIL-101(Cr)/MWCNTs-	Triazines	Corn	HPLC–MS/MS	0.01–0.04	90.3–116.5	1.08–12.32	[71]
Graphene	Sulfonamides	Milk, eggs, and lake water	HPLC-UV	0.03–0.44	90–108	<10.1	[10]
Copper sheets	Sulfonamides	Milk and lake water	HPLC-UV	0.075–0.35 and 0.009–0.019	88–97 and 89–102	6.8–9.9	[11]
Urea–formaldehyde co-oligomers	Fenbufen, flurbiprofen, benzophenone-8, butylparaben, cumylphenol, 4-octylphenol, chlorpyrifos, trifluralin, deltamethrin, tonalide	Lake water	HPLC-UV	0.01–0.33	92–100	5.6–8.4	[12]
β-Cyclodextrin and graphene oxide	Malachite green	Crayfish and squid	HPLC-Vis	0.21	88.6–100.8	-	[70]
Polyaniline	Perfluorooctanoic acid and perfluorooctane sulfonate	Human serum and urine	HPLC/MS	-	79–91	5.5–8.2	[72]
Trichloromethylsilane	Benzene, toluene, ethylbenzene, *m*-xylene, and *o*-xylene	Hookah, gulf water, and petrochemical wastewater	GC/MS	0.005–0.0010	91–105	<13	[73]
Ni–Co layered double hydroxides	Gallic acid, *p*-hydroxybenzoic acid, and caffeic acid	Fruit juices	HPLC-UV	0.15–0.35	89.7–95.3	<10	[74]
Silica monolith	Dipeptides (Tyr–Gly, Phe–Gly, Tyr–Val, Tyr–Ala, 3-I-Tyr–Ala, 3,5-dI-Tyr–Ala)	Water	HPLC–MS/MS	0.00002−0.0013	100	2–3	[75]
